# Severe acute respiratory syndrome coronavirus 2 prevalence in saliva and gastric and intestinal fluid in patients undergoing gastrointestinal endoscopy in coronavirus disease 2019 endemic areas: Prospective cross‐sectional study in Japan

**DOI:** 10.1111/den.13945

**Published:** 2021-03-12

**Authors:** Shigeta Miyake, Keiichi Ashikari, Shingo Kato, Tomohiro Takatsu, Hirofumi Kuwashima, Hiroaki Kaneko, Koki Nagai, Ikue Watari, Takamitsu Sato, Yutaro Yamaoka, Tetsuya Yamamoto, Akihide Ryo, Shin Maeda, Atsushi Nakajima, Takuma Higurashi

**Affiliations:** ^1^ Departments of Neurosurgery Graduate School of Medicine Yokohama City University Kanagawa Japan; ^2^ Gastroenterology and Hepatology Graduate School of Medicine Yokohama City University Kanagawa Japan; ^3^ Gastroenterology Yokohama City University Kanagawa Japan; ^4^ Clinical Cancer Genomics Graduate School of Medicine Yokohama City University Kanagawa Japan; ^5^ Department of Microbiology School of Medicine Yokohama City University Kanagawa Japan; ^6^ Life Science Laboratory, Technology and Development Division Kanto Chemical Co., Inc. Kanagawa Japan

**Keywords:** COVID‐19, endoscopy, gastrointestinal fluid, saliva, SARS‐CoV‐2

## Abstract

**Objectives:**

Gastrointestinal endoscopy (GIE) is useful for the early detection and treatment of many diseases; however, GIE is considered a high‐risk procedure in the coronavirus disease 2019 (COVID‐19) pandemic era. This study aimed to explore the rate of severe acute respiratory syndrome coronavirus 2 (SARS‐CoV‐2) positivity in saliva and gastrointestinal fluids to which endoscopy medical staff are exposed.

**Methods:**

The study was a single‐center cross‐sectional study. From June 1 to July 31, 2020, all patients who underwent GIE at Yokohama City University Hospital were registered. All patients provided 3 mL of saliva. For upper GIE, 10 mL of gastric fluid was collected through the endoscope. For lower GIE, 10 mL of intestinal fluid was collected through the endoscope. The primary outcome was the positive rate of SARS‐CoV‐2 in saliva and gastrointestinal fluids. We also analyzed serum‐specific antibodies for SARS‐CoV‐2 and patients’ background information.

**Results:**

A total of 783 samples (560 upper GIE and 223 lower GIE samples) were analyzed. Polymerase chain reaction (PCR) on saliva samples did not show any positive results in either upper or lower GIE samples. However, 2.0% (16/783) of gastrointestinal fluid samples tested positive for SARS‐CoV‐2. No significant differences in age, sex, purpose of endoscopy, medication, or rate of antibody test positivity were found between PCR positive and PCR negative cases.

**Conclusions:**

Asymptomatic patients, even those with no detectable virus in their saliva, had SARS‐CoV‐2 in their gastrointestinal tract. Endoscopy medical staff should be aware of infection when performing procedures. The study was registered as UMIN000040587.

## Introduction

The coronavirus disease 2019 (COVID‐19) pandemic is currently one of the greatest human challenges facing healthcare and economic systems worldwide.^1^, ^2^, ^3^ COVID‐19, which is caused by severe acute respiratory syndrome coronavirus 2 (SARS‐CoV‐2), has affected more than 27 million individuals and caused more than 0.8 million deaths to date.^4^ The basic routes of coronavirus transmission are droplet and contact transmission, and the main routes of transmission of SARS‐CoV‐2 are believed to be the same.^5^ COVID‐19 is highly transmissible, adequate use of disposable personal protective equipment (PPE) is recommended to prevent outbreaks of COVID‐19 in hospitals.^6^ It is also very important to protect the healthcare system by postponing unnecessary medical visits and tests as much as possible.

Gastrointestinal endoscopy (GIE) is useful for the early detection and treatment of gastrointestinal (GI) cancer, exclusion of organic diseases, and emergency cases, such as GI bleeding. However, upper GIE (esophagogastroduodenoscopy [EGD]) can induce coughing in patients; thus, there is a risk of aerosol transmission to healthcare workers.^7^ Furthermore, it has been suggested that people infected with COVID‐19 may shed the virus in their feces long after they have been cured.^8^ Therefore, there is also a potential risk of infection with lower GIE (colonoscopy [CS]). Patients with confirmed or clinically suspected COVID‐19 are treated as high‐risk patients in endoscopic practice, and GIE is only recommended in emergency cases. Furthermore, even in patients not clinically suspected of having COVID‐19 (low‐risk patients in endoscopic practice), consideration should be given to postponing non‐emergency GIE procedures, including EGD, CS and endoscopic ultrasonography (EUS), because patients with mild cases of COVID‐19 are often asymptomatic, but even asymptomatic patients are infectious.^9^, ^10^ However, the real risk of transmission between endoscopists/examiners with PPE and low‐risk patients has not been established. Nevertheless, it cannot be denied that prolonged interruptions in non‐emergency endoscopy and endoscopic medical checkups may cause serious disadvantages, such as delays in the diagnosis or progression of disease, to patients and examiners. Therefore, it is necessary to consider resuming normal GIE procedures, including endoscopic medical checkups, with appropriate triage and reliable infection control measures.

In Japan, COVID‐19 has affected 67,595 individuals and caused 1295 deaths as of September 1, 2020.^11^ In Japan, a state of emergency for COVID‐19 was declared from April 7 to May 25, 2020. Yokohama City University (YCU) Hospital is a tertiary‐level hospital located in Yokohama City, Kanagawa Prefecture, the second most populous prefecture in Japan after Tokyo with 9.2 million residents. In this period, we postponed non‐emergency GIE procedures. After the state of emergency was lifted, GIE procedures were resumed at our hospital with pre‐examination triage and standard precautions with complete PPE including the N‐95 mask, according to the Japanese Gastroenterological Endoscopy Society (JGES) recommendation. However, in resuming GIE procedures in the current COVID‐19 era, it was necessary to assess the safety of GIE and establish adequate infection control measures to relieve medical professionals working within the endoscopic center.

This study aimed to explore the rate of SARS‐CoV‐2 positivity in saliva, gastric, and intestinal fluids to which endoscopy providers may be exposed.

## Methods

### Study design and setting

The study was a single‐center cross‐sectional study to evaluate the prevalence of SARS‐CoV‐2 positivity in saliva and gastric and intestinal fluids, in patients deemed to be at a low risk of COVID‐19 and who were scheduled to undergo endoscopy at YCU Hospital. From June 1 to July 31, 2020, all patients who underwent GIE at the YCU Hospital were registered.

### Ethical approval and study registration

The institutional review board at YCU Hospital approved this prospective study on May 17, 2020 (approval number: B200500054). Written consent for participation in the study was obtained from all patients. The study was registered in the University Hospital Medical Information Network Clinical Trials Registry as UMIN000040587.

### Participants

To reduce the risk of infection to healthcare providers, patients who underwent GIE were subject to a brief medical check and required to complete a questionnaire. Patients were classified as either COVID‐19 high‐risk or low‐risk patients. Patients who had (i) confirmed COVID‐19; (ii) a history of concentrated contact with a patient or suspected patient with COVID‐19 within 2 weeks; (iii) COVID‐19 related symptoms, such as fever (>37.5°C), dyspnea, or severe malaise; (iv) abnormalities of taste and smell with no obvious triggers; and (v) GI symptoms, such as diarrhea, lasting 4–5 days without obvious triggers, were classed as high‐risk patients. The remaining patients were classified as low‐risk patients.

The inclusion criteria were as follows: (i) low‐risk patients; (ii) non‐emergency GIE cases; (iii) outpatients; (iv) patients who provided written informed consent for study participation.

The exclusion criteria were as follows: (i) high‐risk patients (high‐risk patients were assessed to identify the purpose and urgency of GIE, and if the procedure was judged as a non‐emergency, it was postponed); (ii) emergency cases (in emergency cases, it was difficult to obtain informed consent); (iii) in patients (our hospital conducted universal COVID‐19 screening before permitting patients to enter the hospital, and in‐patient samples had already undergone real‐time reverse transcription polymerase chain reaction [RT‐PCR]); and (iv) patients not willing to participate in the study.

We analyzed baseline characteristics, including age, sex, purpose of endoscopy, and medications.

### Endoscopy and sample collection

In cases of upper GIE, including EGD and EUS, patients were required to fast for at least 12 h before the procedure. Before upper GIE, patients supplied 3 mL of saliva and received adequate sedation without oral dimethicone for antifoam. After insertion into the esophagus, the endoscope was immediately inserted into the stomach and used to vacuum 10 mL of gastric fluid. Then, endoscopists performed usual procedures. In cases of lower GIE, such as CS, bowel preparation was initiated 1 day before the procedure. Each patient was instructed to consume a low‐residue diet and to take 5 mg of oral sodium picosulfate on the evening before the procedure. On the day of the procedure, each patient was administered 1500 mL of polyethylene glycol (PEG). Before the procedure, patients also supplied 3 mL of saliva and received adequate sedation. After insertion into the cecum, the endoscope was used to vacuum 10 mL of intestinal fluid. Then, endoscopists performed usual procedures. Collected saliva and gastric and intestinal fluids were stored at −80°C until analysis. Patients who agreed to undergo additional blood sampling had 5 mL of blood drawn. Collected blood was separated from serum and stored at −80°C until analysis.

### Outcomes

The primary outcome was the positive rate of SARS‐CoV‐2 in saliva and gastric and intestinal fluids. Iwasaki et al. reported that the concordance rate of virus detection between saliva and nasopharyngeal samples was as high as 97.4%.^12^ Following this report, we tested saliva samples, because collecting nasopharyngeal swab samples for testing is highly invasive and a risk of infection to examiners. We also analyzed serum‐specific antibodies for SARS‐CoV‐2. Furthermore, we evaluated patients’ background characteristics, including age, sex, purpose of GIE, and medications.

### Detection of SARS‐CoV‐2 genomic RNA and serological tests for SARS‐CoV‐2 antibodies

Detection of SARS‐CoV‐2 genomic RNA was performed according to the Manual for the Detection of Pathogen 2019‐nCoV Ver.2.6 provided by the National Institute of Infectious Diseases in Japan.^13^ The detailed methods and primer sequences, the methods of serological testing^14^, ^15^ are shown in Text S1 and Table S1.

### Statistical analysis

Results are presented as the mean for quantitative data and frequency (percentage) for categorical data. Categorical data were assessed using the Chi‐squared test. The Mann–Whitney *U*‐test or the student’s *t*‐test was used to compare continuous data. *P*‐values < 0.05 were considered statistically significant. All statistical analyses were performed using JMP 15 software (SAS Institute Inc., Cary, NC, USA).

## Results

### Patient characteristics

**Figure 1:**
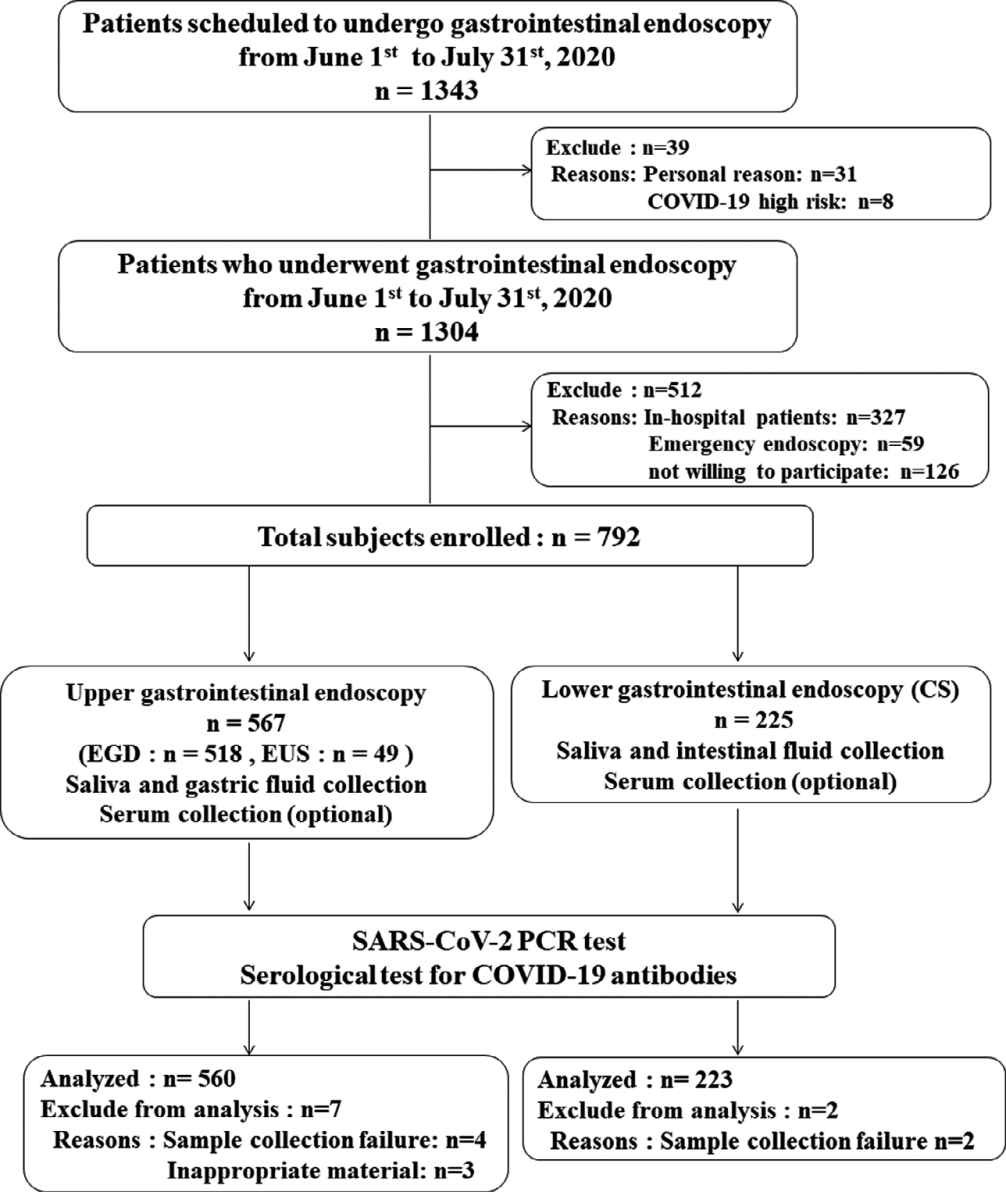
Study flowchart. Between June 1 and July 31, 2020, a total of 1343 patients were scheduled to undergo gastrointestinal endoscopy (GIE). A total of 1304 patients actually underwent GIE at our endoscopy center. A total of 512 patients were excluded because they did not meet the inclusion criteria. The remaining 792 patients (518 patients who underwent esophagogastroduodenoscopy (EGD), 49 patients who underwent endoscopid ultrasound (EUS), and 225 patients who underwent colonoscopy (CS)) consented to participate in the study and supplied samples. Finally, a total of 783 specimens (511 from patients who underwent EGD, 49 from patients who underwent EUS, and 223 from patients who underwent CS) were sent for final analysis.

During the study period (June 1 to July 31, 2020), the number of newly diagnosed patients with COVID‐19 was 1,116 in Kanagawa Prefecture. The prevalence of infected people was 0.46 (per 100,000 per a day) in the last week of July.^16^ During this period, a total of 1343 patients were scheduled to undergo GIE; 31 of these procedures were canceled due to personal reasons and eight were postponed because they were judged as high‐risk for COVID‐19. A total of 1304 patients underwent GIE at our endoscopy center. A total of 512 patients were excluded because they did not meet the inclusion criteria. The reasons for exclusion were as follows: 327 were in‐hospital cases, 59 were emergency endoscopy cases, and 126 were not willing to participate in the study. The remaining 792 patients (518 EGD cases, 49 EUS cases, and 225 CS cases) consented to participate in the study and supplied samples. For the seven patients who underwent EGD, no specimens were obtained or the samples were inappropriate. For the two patients who underwent CS, no specimens were obtained. A total of 783 samples (511 samples from patients undergoing EGD, 49 samples from patients undergoing EUS, and 223 samples from patients undergoing CS) were sent for final analysis (Fig. 1).

The clinical characteristics of patients are presented in Table 1. The mean age was 69.2 ± 11.6 years, and 485 patients (61.9%) were male. The reasons for GIE were as follows: 84.9% (685/783) for screening or follow‐up, 13.3% (104/783) for detailed examination, and 1.7% (13/783) for treatment. A total of 34.9% of patients (273/783) were administered medicine for the stomach, including proton pump inhibitors (PPIs) or vonoprazan (potassium‐competitive acid blocker), and 28.3% of patients (222/783) were administered angiotensin‐converting enzyme (ACE) inhibitors or angiotensin receptor blockers (ARBs). None of the participants demonstrated any COVID‐19‐related symptoms or a history of contact with COVID‐19‐infected patients.

**Table 1:** Patient characteristics

Procedures	EGD	EUS	CS	Total
Number	511	49	223	783
Age, years., (mean ± SD)	70.6 ± 11.2	68.2 ± 10.7	66.3 ± 12.2	69.2 ± 11.6
Sex (male, %)	325 (63.6)	29 (59.2)	131 (58.7)	485 (61.9)
Purpose of endoscopy				
Screening/follow‐up, *n* (%)	452 (88.5)	8 (16.3)	205 (91.9)	665 (84.9)
Detailed examination, *n* (%)	57 (11.2)	41 (83.7)	6 (2.7)	104 (13.3)
Treatment, *n* (%)	2 (3.9)	0 (0)	11 (4.9)	13 (1.7)
Medication				
PPI (including vonoprazan), *n* (%)	192 (37.6)	18 (36.7)	63 (28.3)	273 (34.9)
ACE inhibitor or ARB, *n* (%)	152 (29.7)	13 (26.5)	57 (25.6)	222 (28.3)

ACE, angiotensin‐converting enzyme; ARB, angiotensin receptor blocker; CS, colonoscopy; EGD, esophagogastroduodenoscopy; EUS, endoscopic ultrasound; PPI, proton pump inhibitor.

### Results of RT‐PCR and serological antibody testing for SARS‐CoV‐2

The RT‐PCR testing for SARS‐CoV‐2 in saliva did not show any positive results in patients who underwent EGD, EUS, and CS. However, surprisingly 2.3% of upper GI samples (13/560) were positive, and limited EGD cases, 2.5% (13/511) were positive (Table 2). A total of 1.3% of CS samples (3/223) were positive. The Ct value of all positive patients was between 33 and 35. In all RT‐PCR tests, the negative control was never amplified, and the 50‐copy positive control had a Ct value of 32–33 (see Table S2).

During the study period, 55.2% of patients (432/783) agreed to have additional blood tests performed, including 271 patients who underwent GS, 45 patients who underwent EUS, and 116 patients who underwent CS. A total of 17 patients (3.9%) across all procedures showed a positive result with serum‐specific antibody testing, including 12 patients who underwent GS (4.4%) and five patients who underwent CS (4.3%) (Table 3). There were no positive serum‐specific antibody tests in patients who underwent EUS (see details in Table S3).

Collected saliva and gastric and intestinal fluids were stored at −80°C until analysis and analyzed together at a later date. Therefore, patients who had positive results did not receive any treatment or medication. However, patients were followed up for 2–4 weeks after GIE, and none of them presented with symptoms related to COVID‐19 during the follow‐up period.

**Table 2:** RT‐PCR test for SARS‐CoV‐2

Specimen (number of patients)	Positive RT‐PCR test (*n*)	Percent (%)
Any specimen (783)	16	2.0
Saliva		
All procedures (783)	0	0
EGD (511)	0	0
EUS (49)	0	0
CS (223)	0	0
Gastric fluid		
All upper GIE (560)	13	2.3
EGD (511)	13	2.5
EUS (49)	0	0
Intestinal fluid		
CS (223)	3	1.3

CS, colonoscopy; EGD, esophagogastroduodenoscopy; EUS, endoscopic ultrasound; GIE, gastrointestinal endoscopy; NP, nucleocapsid protein; RT‐PCR, real‐time reverse transcription polymerase chain reaction; SARS‐CoV‐2, severe acute respiratory syndrome coronavirus 2; SP, spike protein.

**Table 3:** Serological test for COVID‐19 antibodies

	Serological test for COVID‐19 antibodies (*n* = 432)	
	Number of positive tests^†^	%
All procedures	17	3.9
EGD	12	4.4
EUS	0	0
CS	5	4.3

COVID‐19, coronavirus disease 2019; CS, colonoscopy; EGD, esophagogastroduodenoscopy; EUS, endoscopic ultrasound; GIE, gastrointestinal endoscopy.

^†^ COVID‐19 antibody positivity was defined as a value above 1.139 in the NP test and above 0.277 in the SP test.

### Endoscopic findings of SARS‐CoV‐2 RT‐PCR positive participants

Among the 13 participants with positive RT‐PCR in gastric fluid, endoscopic findings were as follows: 10 participants had atrophic gastritis, but this had eradicated *Helicobacter pylori* in all participants; two participants had normal findings; and one had remnant gastritis. No participants had redness, edema, erosion or ulcer that were suspected infection in the stomach. There were also no characteristic findings in the pharynx.

Among three patients who were RT‐PCR‐positive in intestinal fluid, none had colitis.

### Comparison of SARS‐CoV‐2 RT‐PCR positive cases and negative cases

We compared patients who were one or more of the samples positive with those who were all negative (Table 4). No significant differences in age, sex, purpose of endoscopy, medications (PPIs and ACE inhibitors or ARBs) or serological antibody titers for SARS‐CoV‐2 were found between SARS‐CoV‐2 RT‐PCR positive and negative patients. As previously described, SARS‐CoV‐2 was not detected in saliva in any patient, but SARS‐CoV‐2 was detected in gastric and intestinal fluids (*P* < 0.001). Of the patients who tested positive for SARS‐CoV‐2 in GI fluid by RT‐PCR, only one patient tested positive for serum antibodies, which did not differ from the percentage of patients in the RT‐PCR‐negative group who tested positive for antibodies.

**Table 4:** Comparison between COVID‐19 PCR positive and negative cases

	COVID‐19 RT‐PCR positive cases (*n* = 16)	COVID‐19 RT‐PCR negative cases (*n* = 767)	*P*‐value^†^
Age, years (mean ± SD)	69.8 ± 12.1	69.2 ± 11.6	0.839
Sex (male, %)	11 (68.8)	474 (61.8)	0.571
Specimen			<0.001
Saliva	0	767	
Gastric fluid	13	547	
Intestinal fluid	3	220	
Purpose			0.598^†^
Screening/follow‐up	15	650	
Detailed examination	1	103	
Treatment	0	14	
Positive COVID‐19 antibody test, number, (%)	1 (6.3)	16 (2.1)	0.258
Antibody titer (NP test)	0.162 ± 0.053	0.150 ± 0.191	0.873
Antibody tier (SP test)	0.117 ± 0.151	0.070 ± 0.076	0.112
Medication			
PPI (including vonoprazan)	5	268	0.759
ACE inhibitor or ARB	2	220	0.155

ACE, angiotensin‐converting enzyme; ARB, angiotensin receptor blocker; COVID‐19, coronavirus disease 2019; NP, nucleocapsid protein; PPI, proton pump inhibitor; RT‐PCR, real‐time reverse transcription polymerase chain reaction; SP, spike protein.

^†^ *P*‐values were calculated using the Chi‐squared test or the student’s *t*‐test between COVID‐19 PCR positive and negative case.

### Medical staff at the endoscopy center

Throughout the study period, no medical staff at the endoscopy center, including endoscopists (*n* = 22), nurses (*n* = 7), receptionists (*n* = 3), and cleaning staff (*n* = 6), developed fever or other health concerns.

## Discussion

In this study, we assessed GI fluid from 783 patients at a low risk of COVID‐19 and demonstrated that 2.5% of gastric fluid samples and 1.3% of intestinal fluid samples tested positive for SARS‐CoV‐2 with RT‐PCR. Serologically, 3.9% of patients tested positive for SARS‐CoV‐2 with serum‐specific antibody testing. However, there was no relationship between RT‐PCR testing of GI fluid and serological testing. Furthermore, no difference in PPI and ACE inhibitor or ARB use was found between patients who tested positive for SARS‐CoV‐2 in GI fluid using RT‐PCR and those who tested negative.

It has been reported that SARS‐CoV‐2 is detected in the GI tract of COVID‐19‐positive individuals and that the virus can be detected in the feces of these patients for some time after it is no longer expelled from sputum or saliva^17^; however, these results were from COVID‐19‐positive individuals. In the present study, we demonstrated absence of SARS‐CoV‐2 in saliva in patients deemed to be at a low risk of COVID‐19 and undergoing endoscopy; however, 2% of patients tested positive for SARS‐CoV‐2 in gastric or intestinal fluids. This suggests that in COVID‐19 endemic areas, a certain percentage of individuals, even asymptomatic individuals, may carry the virus in the GI tract. Furthermore, our results demonstrate that SARS‐CoV‐2 can be present not only in the lower GI tract, but also in the upper GI tract. Under condition of the pandemic, a SARS‐CoV‐2 RT‐PCR test may be recommended for all patients who are scheduled to undergo endoscopy. Furthermore, endoscopy providers had better wear complete PPE during endoscopic procedures.

None of the participants had a history of COVID‐19‐related symptoms, and therefore, it is unclear at which point RT‐PCR‐positive patients became infected. According to the current definition of the World Health Organization, even if the patient is asymptomatic, if their results in a RT‐PCR test are positive, the patient should be treated as infected.^18^ In that sense, these patients who were positive in only GI fluid can be considered as infected. According to previous reports, SARS‐CoV‐2 can be detected in the feces for some time after a patient has recovered from COVID‐19. It is reported that saliva, sputum, and pharyngeal swabs can detect SARS‐CoV‐2 within 1 week of COVID‐19 onset; as time passes, the detection rate declines. However, it is still unknown how long SARS‐CoV‐2 can be detected in GI fluid and whether its presence is infectious. However, cases of infection via aerosols generated from stool in toilets have been reported in other countries.^19^ To our knowledge, there are no cases of SARS‐CoV‐2 infection via endoscopic procedures to date. We followed up with medical staff and patients for 2–4 weeks after GIE, without medication; none of them presented symptoms related to COVID‐19 during the follow‐up period. Identification of SARS‐CoV‐2 in GI fluid is a novel finding, but there are limited data on the length of time the virus remains in GI fluid and how long it remains infectious. Further studies are needed to clarify the characteristics and clinical importance of SARS‐CoV‐2 in the GI tract.

We previously reported that SARS‐CoV‐2 antibodies can be detected approximately 1 week after infection, with almost all cases testing positive after 3 weeks with a gradual decline thereafter.^14^ In this study, we detected 3.9% patients serologically positive for SARS‐CoV‐2. However, only one patient who tested positive on RT‐PCR in GI fluid was positive for serum antibodies test. Furthermore, the remaining 16 patients who tested positive for SARS‐CoV‐2 with the serum antibody test were negative when GI fluid was tested with RT‐PCR. It is possible that patients whose GI fluid tested positive were still within 3 weeks of exposure to SARS‐CoV‐2; thus, their antibodies were not elevated. A second possibility is that the virus in the GI tract was so minimal that it did not cause antibodies to become elevated. Third, patients with a positive antibody test but a negative GI fluid RT‐PCR test may have been exposed to the virus a long time prior to testing.

Previous studies have reported that PPI use is a risk factor for COVID‐19 infection and that this effect is dose‐dependent.^20^ This suggests that gastric acid is a barrier to GI infection in patients with COVID‐19, and suppressing gastric acid secretion with PPIs may increase the risk of infection. However, our results did not reveal the relationship between PPI use and SARS‐CoV‐2 detection in the GI tract. Further analysis is needed to clarify the relationship between PPI use and COVID‐19.

It is reported that SARS‐CoV‐2 invades cells using ACE2 as its receptor.^21^, ^22^ Furthermore, ACE inhibitor/ARB use increases the expression of ACE2 in the GI tract in a rodent model.^23^ We also analyzed the relationship between ACE inhibitors/ARBs and COVID‐19; however, there was no relationship between ACE inhibitor/ARB use and COVID‐19 detection. The European Society of Cardiology and the European Society of Hypertension issued a statement (supported by the International Society of Hypertension) that conventional antihypertensive treatment, including ACE inhibitors/ARBs, should continue.^24^, ^25^, ^26^ Our findings are consequent of these clinical reports.

Our study had several limitations. First, the study was based on our single‐center experience. Our hospital is a tertiary‐level hospital in Yokohama. Thus, patients at our hospital had many comorbidities, and patient bias may be present. However, this study was conducted in a large number of patients; thus, the results are considered reliable. Second, this study is cross‐sectional study with an investigation period of only 2 months. The positive rate of SARS‐CoV‐2 is constantly changing. We should bear in mind the rate of infection in the area. Third, this study was not based on a rigorous sample size calculation. However, COVID‐19 is an emerging infectious disease and the data were limited at that point; therefore, it was difficult to conduct an exact sample size calculation. Fourth, medical providers did not undergo RT‐PCR testing. However, we believe that endoscopy can be performed safely if endoscopy staff are provided adequate protection against infection. Finally, RT‐PCR and antibody tests may contain false positives and false negatives. We did not retest study specimen; retests sometimes show different test results. A recent meta‐analysis reports that in the RT‐PCR test for SARS‐CoV‐2, sensitivity is 89.1% (95%CI 84.0–92.7%) and specificity is 98.9% (95%CI 98.0–99.4%).^27^ However, the true accuracy of RT‐PCR in this study remains unknown, because study participants did not undergo further examination. Despite these limitations, it is clear that a certain percentage of people, even asymptomatic ones, may carry SARS‐CoV‐2 in their GI tract. Further investigation of the RT‐PCR‐positive period and the infectivity of SARS‐CoV‐2 in GI fluid is required.

We conclude that approximately 2.0% of asymptomatic patients, even patients with no detectable virus in their saliva, tested positive for SARS‐CoV‐2 in their GI tract. Endoscopy staff should continue to be aware of the risk of infection when performing procedures. Furthermore, even if people are asymptomatic, people should be careful when handling feces and when using the restroom during the COVID‐19 era. Further studies are needed to clarify the relationship between COVID‐19 and the presence of SARS‐CoV‐2 in the GI tract.

## Conflict of Interest

Authors declare no conflicts of interest for this article.

## Funding Information

Grants for this research from the Japanese Foundation for Research and Promotion of Endoscopy (JFE) and Daiwa Securities Health Foundation were awarded to TH. We declare that the funding body has no role in the design, data collection, analysis, interpretation and writing of the study.

## Supporting information


**Text S1** Detection of SARS‐Co‐V‐2 genomic RNA and serological tests for SARS‐CoV‐2 antibodies.
**Table S1** Sequences of the primers.
**Table S2** CT values in RT‐PCR positive cases.
**Table S3** Serological antibody test results in positive cases.
