# Supplementary material for: Severe acute respiratory syndrome coronavirus 2 prevalence in saliva and gastric and intestinal fluid in patients undergoing gastrointestinal endoscopy in coronavirus disease 2019 endemic areas: Prospective cross‐sectional study in Japan
Source: Dig Endosc. 2021 Mar 12;34(1):96–104. doi: 10.1111/den.13945 (PMC8014498; doi:10.1111/den.13945)
Supplement: Supplementary file 1 — Text S1 Detection of SARS‐Co‐V‐2 genomic RNA and serological tests for SARS‐CoV‐2 antibodies. Table S1 Sequences of the primers. Table S2 CT values in RT‐PCR positive cases. Table S3 Serological antibody test results in positive cases. [file DEN-34-96-s001.docx]

**Supplementary Text 1**

**Detection of SARS-Co-V-2 genomic RNA and serological tests for SARS-CoV-2 antibodies**

Detection of SARS-CoV-2 genomic RNA was performed according to the Manual for the Detection of Pathogen 2019-nCoV Ver.2.6 provided by the National Institute of Infectious Diseases in Japan.^(1)^ RNA extraction was performed from a 140-µl sample using a QIAamp Viral RNA Mini Kit (Qiagen, Valencia, CA, USA) according to the manufacturer’s protocol. The final elution was performed with 60 µl of elution buffer, and 5 µl of extracted RNA was subject to evaluation by real-time quantitative PCR (RT-qPCR). The RT-qPCR reaction mix was prepared using TaqMan Fast Virus 1-Step Master Mix (Thermo Fisher Scientific, Foster City, CA, USA) and primer/probe N2 (2019-nCoV) (TakaRa, Tokyo, Japan) according to the manufacturer’s protocol. Primer sequences are shown in Supplementary Table 1. RT-PCR was conducted using the StepOnePlus^TM^ Real-Time PCR System (Thermo Fisher Scientific, MA, USA) The denaturation and annealing/extension steps were repeated for 45 cycles. A well containing absolutely quantified artificial synthetic template RNA was evaluated as a positive control, and a well without template RNA was evaluated as a negative control. According to the Manual for the Detection of Pathogen 2019-nCoV Ver.2.6, the assay was considered valid when the following criteria were met: (a) 50 copies/well of template RNA successfully detected before 40 cycles and (b) nonspecific amplification not detected in the well lacking template RNA up to 45 cycles. The cut-off value is automatically determined according to the increase in the positive control in each PCR test. Samples showing amplification of SARS-CoV-2 genomic RNA before 40 cycles were defined as positive for COVID-19. The appropriate cut-off values are unknown for gastric and intestinal fluid. Therefore, we adopted the same cut-off value to GI fluid.

We previously reported a method for serological testing.^(2, 3)^ Briefly, an enzyme-linked immunosorbent assay was performed to detect and quantify anti-SARS-CoV-2 antibodies in plasma. We used an N-terminally-truncated nucleocapsid protein (NP) and the receptor-binding domain of the spike protein (SP) as antigens (100 ng/well). Following blocking with phosphate-buffered saline (PBS) containing 3% skimmed milk for 2 h, 100 µl of diluted plasma (1:100) were added and incubated for 1 h. After three washes with PBS-T, wells were incubated with 100 µl of diluted horseradish peroxidase-conjugated goat anti-human immunoglobulin G antibody (1:20000) for 1 h. After five additional washes with PBS-T, 100 µl of Tetramethylbenzidine Substrate (Kirkegaard & Perry Laboratories, Maryland, USA) was added and incubated for 10 mins. The reaction was terminated by adding 50 µl of 2-M H_2_SO_4_, and optical density was measured at 450 nm using a plate reader. In the serological test, COVID-19 antibody positivity was defined as a value greater than 1.139 in the NP test and greater than 0.277 in the SP test. It has been reported that antibodies to nucleocapsid protein (NP) tend to arise earlier after infection than those to spike protein (SP),^(4)^ and that antibody titers to SP tend to be maintained whereas those to NP tend to decline.^(5)^ In this study, a positive result in either the NP test or the SP test was defined as a positive antibody test.

References

1. National Institute of Infectious Diseases J. Manual for the Detection of Pathogen 2019-nCoV Ver.2.6 [cited 2020 February 17]. Available from: <https://www.niid.go.jp/niid/images/epi/corona/2019-nCoVmanual20200217-en.pdf>.

2. Sethuraman N, Jeremiah SS, Ryo A. Interpreting Diagnostic Tests for SARS-CoV-2. Jama. 2020;323(22):2249-51.

3. Yamaoka Y, Jeremiah SS, Miyakawa K, Saji R, Nishii M, Takeuchi I, et al. Whole nucleocapsid protein of SARS-CoV-2 may cause false positive results in serological assays. Clin Infect Dis. 2020:ciaa637.

4. Borremans B, Gamble A, Prager KC, Helman SK, McClain AM, Cox C, et al. Quantifying antibody kinetics and RNA detection during early-phase SARS-CoV-2 infection by time since symptom onset. Elife. 2020;9:e60122.

5. Ripperger TJ, Uhrlaub JL, Watanabe M, Wong R, Castaneda Y, Pizzato HA, et al. Orthogonal SARS-CoV-2 Serological Assays Enable Surveillance of Low-Prevalence Communities and Reveal Durable Humoral Immunity. Immunity. 2020;53(5):925-33.e4.

Supplementary Table 1. Sequences of the primers

| Name | Sequence (5′ to 3′) | Position | Concentration |
| --- | --- | --- | --- |
| NIID_2019-nCOV_N_F2 | AAATTTTGGGGACCAGGAAC | 29142–29161 | 500 nM |
| NIID_2019-nCOV_N_R2 | TGGCAGCTGTGTAGGTCAAC | 29299–29280 | 700 nM |
| NIID_2019-nCOV_N_P2 | FAM-ATGTCGCGCATTGGCATGGA-BHQ | 29239–29258 | 200 nM |

Supplementary Table 2. Ct values in RT-PCR positive cases

|  | sample | Ct value | Copy number | threshold line | Ct value of positive control (50 copy) | Ct value of positive control (500 copy) |
| --- | --- | --- | --- | --- | --- | --- |
| 1 | EGD135 | 35.188 | 6.956 | 0.0214 | 32.153 | 28.257 |
| 2 | EGD139 | 34.986 | 7.128 | 0.0214 | 32.153 | 28.257 |
| 3 | EGD205 | 34.808 | 6.025 | 0.0355 | 32.592 | 28.946 |
| 4 | EGD206 | 34.849 | 5.988 | 0.0355 | 32.592 | 28.946 |
| 5 | EGD210 | 34.807 | 6.026 | 0.0355 | 32.592 | 28.946 |
| 6 | EGD211 | 33.908 | 6.845 | 0.0355 | 32.592 | 28.946 |
| 7 | EGD212 | 34.845 | 5.992 | 0.0355 | 32.592 | 28.946 |
| 8 | EGD215 | 34.700 | 6.124 | 0.0355 | 32.592 | 28.946 |
| 9 | EGD217 | 34.591 | 6.223 | 0.0355 | 32.592 | 28.946 |
| 10 | EGD218 | 33.920 | 6.834 | 0.0355 | 32.592 | 28.946 |
| 11 | EGD220 | 34.945 | 5.900 | 0.0355 | 32.592 | 28.946 |
| 12 | EGD222 | 34.979 | 5.870 | 0.0355 | 32.592 | 28.946 |
| 13 | EGD227 | 34.871 | 12.303 | 0.0348 | 33.627 | 29.073 |
| 14 | CS188 | 34.625 | 12.483 | 0.0348 | 33.627 | 29.073 |
| 15 | CS199 | 35.861 | 7.402 | 0.0348 | 33.627 | 29.073 |
| 16 | CS218 | 35.258 | 7.912 | 0.0376 | 32.965 | 29.038 |

Ct: cycle threshold, EGD: esophagogastroduodenoscopy, CS: colonoscopy, RT-PCR: real-time reverse transcription polymerase chain reaction.

Supplementary Table 3. Serological antibody test results in positive cases

| Sample ID | NP test | SP test |
| --- | --- | --- |
| EGD38 | 0.079 | 0.303 |
| EGD 50 | 0.083 | 0.456 |
| EGD 53 | 1.303 | 0.041 |
| EGD 144 | 0.599 | 0.325 |
| EGD 180 | 0.087 | 0.704 |
| EGD 188 | 0.064 | 0.341 |
| EGD 244 | 0.506 | 0.458 |
| EGD 258 | 0.059 | 0.347 |
| EGD 272 | 1.566 | 0.052 |
| EGD 300 | 0.539 | 0.516 |
| EGD 357 | 0.103 | 0.305 |
| EGD 395 | 0.350 | 0.372 |
| CS72 | 0.119 | 0.341 |
| CS114 | 0.512 | 0.339 |
| CS119 | 2.533 | 0.296 |
| CS188 | 0.102 | 0.450 |
| CS192 | 0.151 | 0.321 |

EGD: esophagogastroduodenoscopy, CS: colonoscopy, NP: nucleocapsid protein, SP: spike protein.

In the serological tests, COVID-19 antibody positivity was defined as a value greater than 1.139 in the NP test and greater than 0.277 in the SP test. A positive result in either the NP test or the SP test was defined as a positive antibody test.
